# A study on the effects of four reading methods on college students’ cognitive abilities and mental health based on positive psychology: taking Lao She’s works as an example

**DOI:** 10.3389/fpsyg.2025.1437790

**Published:** 2025-09-12

**Authors:** Yamei Liu

**Affiliations:** School of Languages and Cultures, Shanghai University of Political Science and Law, Shanghai, China

**Keywords:** positive psychology, reading methods, college students, mental health, cognitive function, anxiety and depression

## Abstract

**Background:**

Rising anxiety and depression among college students demand effective interventions. While reading benefits mental health, the comparative effects of different reading methods (paper, digital, audio, video) remain unclear.

**Methods:**

Two thousand eight hundred sixty students were screened (SAS/SDS) and randomized into paper (PTR), digital (ETR), audio (ALR), video (VIR), or control groups. All groups read Lao She’s works with positive psychology themes for 8 weeks. Cognitive (CFQ, BRIEF-A) and mental health (SAS/SDS) outcomes were measured.

**Results:**

PTR and ALR most effectively improved cognitive function (reduced CFQ scores) and mental health (lower anxiety/depression). ETR showed modest benefits, while VIR had minimal impact. The control group showed no improvement.

**Conclusion:**

Paper and audio reading are optimal for enhancing cognition and mental health in students, offering practical guidance for evidence-based interventions.

## 1 Introduction

The increasing prevalence of negative emotions, such as anxiety and depression, among college students has raised significant concerns regarding their cognitive functioning and mental health (Zvolensky et al., 2020). Academic stress, interpersonal challenges, and uncertain career prospects contribute to heightened psychological vulnerability in this population (Zu et al., 2020; Zuo et al., 2025; Zou et al., 2020). Research indicates that poor mental health in higher education has become a critical public health issue, with severe consequences including impaired cognitive performance and increased risk of self-harm (Zou et al., 2018; Zou et al., 2021).

Mental health difficulties have been shown to negatively impact cognitive abilities, including attention, memory, and learning efficiency. Studies suggest that anxiety and depression reduce life satisfaction, diminish engagement in daily activities, and impair academic performance (Zwick and Wolkenstein, 2017). Furthermore, poor mental health is associated with disrupted sleep quality, which exacerbates cognitive deficits such as poor concentration and reduced information retention (Zweerings et al., 2019). Chronic negative emotions also contribute to physiological stress responses, increasing susceptibility to physical health issues such as headaches, gastrointestinal disturbances, and weakened immune function (Zvolensky et al., 2016; Zu et al., 2025). Additionally, depression and anxiety have been linked to decreased motivation, impaired decision-making, and reduced participation in meaningful activities, further worsening cognitive and psychological well-being (Zu et al., 2014; Zuroff et al., 2000).

Positive psychology offers a promising approach to mitigating these challenges by fostering positive emotions, resilience, and well-being. Interventions based on positive psychology principles have been found to reduce depressive symptoms and enhance psychosocial functioning (Basurrah et al., 2022; Park, 2015). Notably, literature-based interventions, particularly those incorporating positive psychological themes, have been shown to improve emotional regulation, self-identity, and social connectedness (Leung et al., 2019; Arslan et al., 2022). Reading positive literary works has also been associated with improved mental health outcomes, including reduced depressive symptoms and enhanced well-being (Liu, 2024; Hanson, 2019).

However, with the diversification of reading methods—such as paper-based, e-reading, audio, and video formats—the cognitive and psychological effects of different reading modalities remain underexplored (Dimitropoulou et al., 2025). While existing research highlights the benefits of literature in promoting mental health, little is known about how various reading methods influence cognitive abilities and psychological well-being. Therefore, this study examines the effects of four reading methods (paper, digital, audio, and video) based on positive psychology, using Lao She’s works as an example, to assess their impact on college students’ cognitive functioning and mental health. The findings aim to provide evidence-based recommendations for optimizing reading-based interventions to support students’ cognitive and psychological well-being.

## 2 Participants and methods

### 2.1 Participants

This study began with subject selection. We conducted the survey by distributing the Self-Assessment Anxiety Scale (SAS) and Self-Assessment Depression Scale (SDS) to current students in colleges and universities in the Shanghai area and utilizing Questionnaire Star. We conducted the questionnaire survey for four universities in Shanghai area, distributed 2,915 questionnaires, and finally collected 2,860 valid questionnaires (of which invalid questionnaires were mainly excluded because of routine filling in errors or incomplete information, etc.). And through the first round of surveys those with both anxiety and depression levels greater than normal were included in the later interventions of this experiment. For ethical reasons, this study did not allow participants with severe mental illness to participate in our experimental intervention (**Table** **1**). Prior to the start of the intervention program, all participants were given a detailed description of the experimental procedure, purpose, and other relevant information. All participants signed an informed consent form and promised that their survey data and related information would be used only for the purpose of this study. In addition, all of our research procedures were approved by the Research Ethics Committee of Shanghai University of Political Science and Law (NO. SH20240126).

**Table 1:** First round of scale surveys.

Statistics	Send out	Strike out	Recall	Validity	Recovery rates (%)	SAS and SAS ≥ 50
(n)	2,915	55	2,860	2,860	98%	820

### 2.2 Experimental design

**Figure 1:**
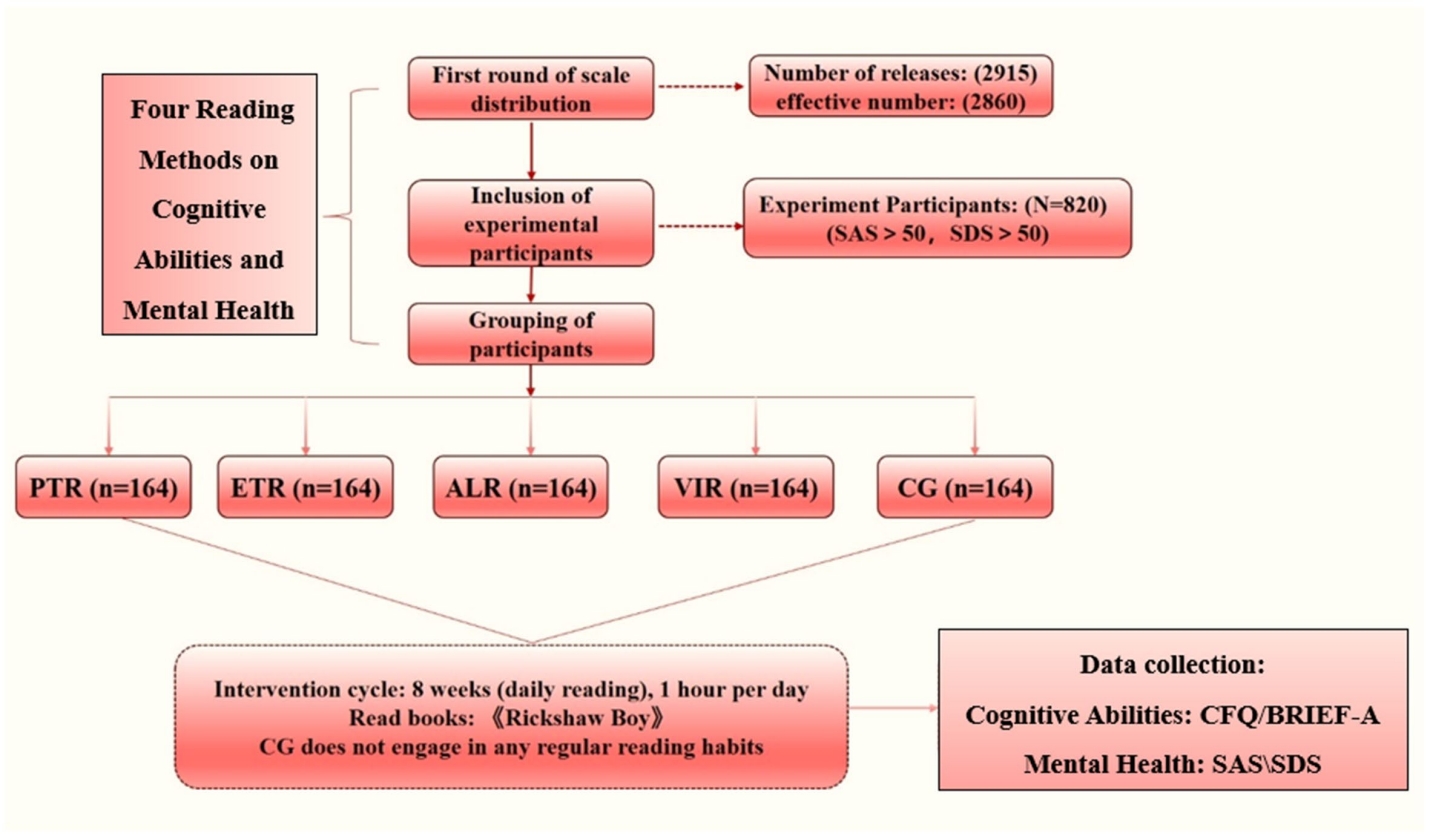
Illustrates the experimental flowchart, detailing the step-by-step process from participant screening to final data analysis. Key stages include initial screening using SAS/SDS scales, randomization into five groups (PTR, ETR, ALR, VIR, and CG), an 8-week intervention with Lao She’s literary works, and pre−/post-test assessments of cognitive function (CFQ, BRIEF-A) and mental health (SAS, SDS). The diagram highlights rigorous design elements such as randomization, controlled intervention, and standardized measurements, ensuring methodological transparency. Abbreviations and visual cues (e.g., group labels, arrows) align with the manuscript for consistency. This flowchart underscores the study’s validity by explicitly mapping adherence to ethical protocols (e.g., informed consent) and analytical rigor (SPSS-based comparisons).

College students suffering from anxiety and depression levels were randomized in equal proportions into five groups (Paper text reading), (Electronic text reading), (Audio listening reading), (Video image reading), (Control group) (**Table** **2**) and an 8-week intervention of literary reading with stories selected from Lao She’s Camel Xiangzi, one of the famous works of modern literature. The stories were selected based on three criteria; (1) positive psychology themes, and (2) participants were given a total of eight chapters (1 per week). Each story contained at least one positive psychology theme, but some stories contained more than one theme. These themes included gratitude, compassion, character strengths, positive thinking, empathy, forgiveness, responsibility, humility, perseverance, and justice. The specific experimental process and protocol are shown in **Figure** 1.

**Table 2:** Statistical grouping of subjects’ information (Mean ± SD).

	PTR	ETR	ALR	VIR	CG
*n*	164	164	164	164	164
Anxiety score	58.21 ± 4.42	59.11 ± 4.37	58.45 ± 3.46	59.29 ± 2.15	58.81 ± 4.17
Depression score	59.67 ± 4.07	59.47 ± 4.01	58.38 ± 3.35	58.29 ± 4.52	58.26 ± 3.92

PTR: paper text reading, ETR: electronic text reading, ALR: audio listening reading, VIR: video image reading, CG: control group.

### 2.3 Methods

#### 2.3.1 Cognitive function scale

##### 2.3.1.1 Cognitive failures questionnaire (CFQ)

The Cognitive Failures Questionnaire (CFQ) was developed by Broadbent (Broadbent et al., 2011) to assess the frequency of everyday cognitive lapses related to attention, memory, and action slips in healthy individuals. The scale consists of 25 self-report items that measure failures in perception, memory, and motor function over the past 6 months. Each item is rated on a 5-point Likert scale (0 = “never” to 4 = “very often”), with total scores ranging from 0 to 100. Higher scores indicate greater cognitive failures, reflecting difficulties in concentration, forgetfulness, and distractibility. Studies have suggested that a CFQ score ≥43 may indicate significant cognitive impairment in daily functioning. The scale has demonstrated good reliability and validity, with a test–retest reliability of 0.82 and a Cronbach’s alpha of 0.88 in this study.

##### 2.3.1.2 Behavior rating inventory of executive function-adult (BRIEF-A)

The Behavior Rating Inventory of Executive Function-Adult (BRIEF-A), developed by a standardized self-report measure assessing executive function (EF) in adults. The scale comprises 75 items across nine clinical subscales, including Inhibition, shifting (cognitive flexibility), Emotional Control, Self-Monitoring, Initiation, Working Memory, Planning/Organization, Task Monitoring, and Organization of Materials. These subscales are further grouped into two composite indices:

Behavioral Regulation Index (BRI) – Measures impulse control and emotional regulation.

Metacognition Index (MI) – Assesses higher-order cognitive functions like planning and working memory.

Each item is rated on a 3-point scale (1 = “never” to 3 = “often”), with higher scores indicating greater executive dysfunction. A Global Executive Composite (GEC) score is derived from all subscales, where a T-score ≥ 65 suggests clinically significant EF impairment. The BRIEF-A has strong psychometric properties, with a test–retest reliability of 0.85 and a Cronbach’s alpha of 0.91 in this study.

#### 2.3.2 Mental health measurement modalities

##### 2.3.2.1 Self-rating anxiety scale (SAS)

The Self-Rating Anxiety Scale (SAS) was developed by William Zung in 1971 (42) and is intended to be widely used among college students for the assessment of anxiety states. The scale consists of 15 positively rated items and 5 negatively rated items. Each item is rated on a four-point scale: 1 for “none or very little of the time”; 2 for “some of the time”; 3 for “most of the time”; and 4 for “The main statistical measure of the SAS is the total score (standardized score).” The total score is calculated by adding the scores of all items, multiplying by 1.25, and rounding to the nearest whole number to obtain the standardized score. Standard scores below 50 are considered normal, while higher standard scores indicate more severe anxiety symptoms. The reliability of the scale was good, with a retest reliability of 0.82. The Cronbacha’s alpha for this study’s scale was 0.976.

##### 2.3.2.2 Self-rating depression scale (SDS)

The Self-Rating Depression Scale (SDS) was originally created by. It consists of 10 positively rated items and 10 negatively rated items. Each item is rated on a four-point scale: 1 for “none or very little of the time”; 2 for “some of the time”; 3 for “most of the time”; 4 for “most or all of the time”; and 5 for “most or all of the time.” “The main statistical indicator of the SDS is the total score.” The crude score was multiplied by 1.25 and rounded to the nearest whole number to generate a standardized score, where the crude score was obtained by adding up the scores of all items in the questionnaire. Standard scores below 50 were considered normal, while higher standard scores indicated more severe depressive symptoms. The reliability of the scale was good with a retest reliability of 0.83. The Cronbacha’s alpha for this study scale was 0.82.

### 2.4 Statistical analysis

SPSS 26.0 software was used for data processing and analysis. All indicators were analyzed by descriptive statistics and expressed using the mean plus or minus standard deviation. Paired-samples t-test was used for within-group comparisons before and after the intervention, while comparisons between groups were analyzed by ANOVA using the difference. The difference value is also the value of the change after the intervention minus the change before the intervention in different reading styles, so that the statistics better reflect the changes brought about by the effect of the intervention between groups, and the significance level was set at 0.05 in the statistical analysis, with a *p*-value of less than 0.05 being regarded as significant.

## 3 Results

### 3.1 Cognitive executive function outcomes before and after 4 literary reading interventions based on positive psychology

Table 3 shows the within-group changes in CFQ and BRIEF-A scores before and after the intervention across the four reading styles. The results indicate that:

**Table 3:** Within-group comparison of four reading styles on CFQ and BRIEF-A (Mean ± SD).

Variant	Groups	Pre-test	Post-test	*t*	*P*
CFQ	PTR	45.32 ± 5.21	38.15 ± 4.87	−18.224	0.000
	ETR	44.87 ± 4.93	39.02 ± 3.76	−14.876	0.000
	ALR	45.10 ± 5.44	37.89 ± 4.52	−20.551	0.000
	VIR	44.95 ± 4.68	39.45 ± 3.91	−16.332	0.000
	CG	45.21 ± 5.03	44.98 ± 4.85	−0.412	0.683
BRIEF-A	PTR	142.50 ± 8.34	132.75 ± 7.62	−25.441	0.000
	ETR	143.20 ± 9.15	134.60 ± 8.43	−22.117	0.000
	ALR	141.85 ± 8.72	131.90 ± 7.85	−28.336	0.000
	VIR	142.30 ± 7.95	133.45 ± 8.12	−19.874	0.000
	CG	143.05 ± 8.50	142.80 ± 8.25	−0.587	0.562

*p* < 0.05 indicates a significant difference; *P* < 0.01 indicates a highly significant difference. CFQ: Cognitive Failures Questionnaire (lower = better), BRIEF-A: Behavior Rating Inventory of Executive Function-Adult (lower = better). PTR: Paper Text Reading, ETR: Electronic Text Reading, ALR: Audio Listening Reading, VIR: Video Image Reading, CG: Control Group.

CFQ scores (reflecting cognitive lapses) significantly improved (lower scores indicate better function) after intervention in the PTR, ETR, ALR, and VIR groups (*p* < 0.01), while the CG showed no significant change (*p* > 0.05).

BRIEF-A scores (reflecting executive function) also significantly improved (lower scores indicate better function) after intervention in the four reading-style groups (*p* < 0.01), with no significant change in the CG (*p* > 0.05).

#### 3.1.1 Between-group differences in intervention effects

Table 4 compares the post-intervention differences between groups. Key findings:

**Table 4:** Between-group comparison of four reading styles on CFQ and BRIEF-A (Mean ± SD).

Variant	Group 1	Group 2	Mean Difference	*P*
CFQ	PTR	ETR	−0.87	0.012
		ALR	0.26	0.621
		VIR	−1.30	0.003
		CG	−6.83	0.000
	ETR	PTR	0.87	0.012
		ALR	1.13	0.008
		VIR	−0.43	0.287
		CG	−5.96	0.000
	ALR	PTR	−0.26	0.621
		ETR	−1.13	0.008
		VIR	−1.56	0.001
		CG	−7.09	0.000
BRIEF-A	PTR	ETR	1.85	0.105
		ALR	0.85	0.402
		VIR	−0.70	0.514
		CG	−10.05	0.000
	ETR	PTR	−1.85	0.105
		ALR	−1.00	0.320
		VIR	−2.55	0.038
		CG	−11.90	0.000

*P* < 0.05 indicates a significant difference and *P* < 0.01 indicates a highly significant difference. PCS: Physical composite score, MCS: mental Composite Score, PTR: paper text reading, ETR: electronic text reading, ALR: audio listening reading, VIR: video image reading, CG: control group.

CFQ Scores:

All four reading styles (PTR, ETR, ALR, VIR) showed significant improvements compared to the CG (*p* < 0.01).

PTR and ALR had a more pronounced effect than ETR and VIR (*p* < 0.05).

BRIEF-A Scores:

All intervention groups outperformed the CG (*p* < 0.01).

PTR and ETR showed comparable effects, as did ALR and VIR (*p* > 0.05 for pairwise comparisons within these pairs).

### 3.2 Mental health outcomes before and after 4 literary reading interventions based on positive psychology

Table 5 shows the within-group changes in anxiety and depression among college students before and after the intervention. Key findings:

**Table 5:** Within-group comparison of four reading styles on mental health (Mean ± SD).

Variant	Groups	Pre-test	Post-test	*t*	*P*
Anxiety	PTR	57.85 ± 4.51	50.92 ± 3.48	7.243	0.000
	ETR	58.97 ± 4.22	57.10 ± 4.25	10.512	0.061
	ALR	57.63 ± 3.38	52.41 ± 3.30	35.207	0.009
	VIR	58.14 ± 2.24	55.83 ± 3.05	10.884	0.043
	CG	57.72 ± 4.05	57.91 ± 5.12	2.741	0.138
Depression	PTR	58.92 ± 4.15	51.73 ± 3.22	36.801	0.000
	ETR	58.63 ± 4.12	55.15 ± 4.38	9.327	0.028
	ALR	57.45 ± 3.41	51.39 ± 4.11	26.449	0.000
	VIR	57.64 ± 4.47	56.28 ± 3.34	11.893	0.085
	CG	57.83 ± 3.87	58.12 ± 4.62	−2.301	0.408

*P* < 0.05 = significant; *p* < 0.01 = highly significant. PTR: Paper Text Reading; ETR: Electronic Text Reading; ALR: Audio Listening Reading; VIR: Video Image Reading; CG: Control Group.

Anxiety:

PTR and ALR groups showed very significant reductions (*p* < 0.01).

VIR had a significant reduction (*p* < 0.05).

ETR and CG showed no significant changes (*p* > 0.05).

Depression:

PTR and ALR had very significant improvements (*p* < 0.01).

ETR showed a significant improvement (*p* < 0.05).

VIR and CG had no significant changes (*p* > 0.05).

Between-Group Differences in Intervention Effects.

Table 6 compares post-intervention differences between groups:

**Table 6:** Between-group comparison of four reading styles on mental health (Mean ± SD).

Variant	Group 1	Group 2	Mean difference	*P*
Anxiety	PTR	ETR	−4.25	0.000
		ALR	−1.82	0.000
		VIR	−3.68	0.000
		CG	−6.29	0.000
	ETR	PTR	4.25	0.000
		ALR	2.43	0.000
		VIR	0.57	0.081
		CG	−2.04	0.000
	ALR	PTR	1.82	0.000
		ETR	−2.43	0.000
		VIR	−1.86	0.000
		CG	−4.47	0.000
Depression	PTR	ETR	−2.72	0.000
		ALR	0.51	0.039
		VIR	−2.85	0.000
		CG	−6.01	0.000
	ETR	PTR	2.72	0.000
		ALR	3.23	0.000
		VIR	−0.13	0.492
		CG	−3.29	0.000
	ALR	PTR	−0.51	0.039
		ETR	−3.23	0.000
		VIR	−3.36	0.000
		CG	−6.52	0.000

*P* < 0.05 indicates a significant difference and *P* < 0.01 indicates a highly significant difference. PCS: Physical composite score; MCS: mental Composite Score; PTR: paper text reading; ETR: electronic text reading; ALR: audio listening reading; VIR: video image reading; CG: control group.

Anxiety:

All interventions outperformed CG (*p* < 0.05).

PTR > ALR > ETR ≈ VIR (no difference between ETR/VIR, *p* > 0.05).

Depression:

All interventions outperformed CG (*p* < 0.05).

ALR > PTR > ETR ≈ VIR (no difference between ETR/VIR, *p* > 0.05).

## 4 Discussion

### 4.1 A positive psychology-based analysis of 4 reading styles affecting college students’ mental health

The results of this study indicate that all four reading intervention methods based on positive psychology (PTR, ETR, ALR, and VIR) significantly improve college students’ cognitive executive function, as evidenced by significant reductions in CFQ (cognitive errors) and BRIEF-A (executive function) scores. Among these, PTR and ALR were most effective in reducing cognitive errors, while the four reading methods showed relatively balanced effects on improving executive function. This finding is consistent with previous research on the impact of reading methods on cognitive function. Some studies have shown that paper-based reading, due to its tactile feedback and spatial localization advantages, can promote deeper cognitive processing, which aligns with the optimal performance of PTR in this study. Additionally, audio reading has been shown to enhance cognitive performance through the emotional transmission of sound and multisensory stimulation (Zatorre et al., 2007). However, contrary to some studies suggesting that e-reading significantly impairs cognitive function, this study found that ETR still resulted in notable improvements in executive function, which may be attributed to the positive psychology-based content design used in the study.

From a mechanism perspective, the advantages of PTR and ALR may stem from their unique cognitive processing characteristics. PTR promotes working memory engagement through tactile-visual multimodal integration, while ALR reduces cognitive load by freeing up visual attention resources through the auditory channel. This aligns with the multi-component theory of executive function, suggesting that different reading methods may influence different aspects of cognitive function through distinct pathways. Notably, although VIR had relatively weaker effects on improving cognitive errors, its effects on enhancing executive function were comparable to those of ALR. This may suggest that while video reading carries the risk of attention distraction, its rich audiovisual stimuli can still effectively activate the executive control network (Huang, 2010). This finding provides new evidence for multimedia learning theory, indicating that under specific content design, video media can also serve as an effective cognitive intervention tool. The results of this study have important implications for practice. First, PTR and ALR can be prioritized as intervention methods to improve college students’ cognitive function. Second, although ETR and VIR are slightly less effective, they still have application value in digital learning environments, especially when combined with positive psychology content design. Future research can further explore the correspondence between different reading methods and specific cognitive function components, as well as the determination of optimal intervention duration (Liu et al., 2021).

The results of this study indicate that the four reading intervention methods based on positive psychology have differentiated effects on college students’ mental health. In terms of anxiety improvement, PTR and ALR showed the most significant effects, followed by VIR, while ETR did not show significant improvement; in terms of alleviating depressive symptoms, ALR and PTR were the most effective, followed by ETR, while VIR also did not produce a significant effect. These findings reveal the distinct characteristics of different reading media in terms of their effects on mental health interventions. This result is consistent with previous research findings on the mechanisms underlying the effects of reading therapy. Some studies have shown that paper-based reading, through the integration of tactile and visual multisensory experiences, can generate stronger immersion and emotional resonance, which aligns with the superior performance of PTR in improving anxiety and depression in this study. Meanwhile, audio reading activates emotional processing brain regions through the emotional transmission of sound and changes in intonation, explaining ALR’s prominent effects in depression interventions. However, contrary to some studies suggesting that video media have the best emotional arousal effects, VIR’s intervention effects were relatively limited in this study, possibly due to the excessive cognitive resource demands of video content. From a mechanism perspective, the advantages of PTR and ALR may stem from their unique multisensory integration characteristics. PTR enhances emotional engagement through tactile feedback, while ALR directly activates emotional centers through paralinguistic cues (e.g., intonation, rhythm) (Zupan et al., 2016; Zupan and Eskritt, 2022). This aligns with the dual-process theory of emotion regulation, suggesting that different reading methods may influence emotional states through distinct neural pathways. Notably, while ETR shows limited efficacy in alleviating anxiety, it still exerts some effect in mitigating depressive symptoms, which may be attributed to the convenience and accessibility of electronic reading, making it a valuable supplement to traditional reading methods.

The findings of this study offer important implications for mental health intervention practices. First, PTR and ALR can be prioritized as intervention methods to address emotional issues among college students, particularly in scenarios requiring rapid alleviation of anxiety symptoms. Second, although VIR has overall weaker effects, it may still have application value in specific populations (e.g., visual learners). Future research could further explore the matching relationship between different reading methods and specific emotional symptoms, as well as the moderating effects of individual differences (e.g., personality traits, cognitive styles) on intervention outcomes.

### 4.2 Limitations of the study

This study has several limitations that should be acknowledged. First, the sample consisted solely of college students in Shanghai, potentially limiting the generalizability of findings to other regions with differing cultural, economic, or educational contexts. Although the total sample size was large (2,860 valid responses), the subgroup analysis involved smaller groups (164 per condition), which may reduce statistical power and obscure meaningful differences. Second, the intervention relied on a single literary work (“Camel Xiangzi”), and its themes may not resonate equally with all students; more contemporary or diverse texts might yield different effects. The eight-week intervention period may also be insufficient to assess long-term impacts on mental health and quality of life. Third, the reliance on self-report scales introduces subjectivity, as responses could be influenced by social desirability bias or varying interpretations of questions. Objective measures (e.g., physiological data for sleep quality) could strengthen future research. Finally, the study did not account for participants’ pre-existing reading habits, abilities, or external factors (e.g., academic stress, life events) that might confound the intervention’s effects. These limitations highlight the need for broader samples, longer-term studies, diversified interventions, and more robust measurement approaches in future research.

### 4.3 Practical application of research

The findings of this study offer actionable insights for educators, mental health professionals, and policymakers seeking evidence-based strategies to support college students’ cognitive and emotional well-being. Given the superior efficacy of paper-based (PTR) and audio-based (ALR) reading interventions, universities could integrate these methods into counseling programs, stress-management workshops, or even classroom curricula—particularly for students exhibiting anxiety or cognitive difficulties. For instance, libraries or student wellness centers might provide curated audiobooks with positive psychology themes or structured paper-reading circles. Meanwhile, the modest benefits of digital reading (ETR) suggest that e-platforms (e.g., educational apps) should prioritize minimizing distractions and embedding interactive elements to enhance engagement. These interventions are scalable, low-cost, and culturally adaptable, making them viable for diverse institutional settings. Additionally, the results caution against overreliance on video-based content (VIR) for mental health interventions, highlighting the need for tailored media selection based on desired outcomes. By aligning reading modalities with specific cognitive or emotional goals, this research empowers stakeholders to design targeted, multimodal support systems for student well-being.

## 5 Conclusion

This study systematically examined the effects of four reading intervention methods based on positive psychology on college students’ cognitive functions and mental health, and reached the following main conclusions: Different reading media exhibit significant differences in improving cognitive executive functions and mental health. Among them, paper-based reading (PTR) and audio reading (ALR) are most effective in reducing cognitive errors and alleviating anxiety and depression symptoms, which is closely related to their unique multisensory integration mechanisms; Electronic reading (ETR) still has some effect in improving executive function and depressive symptoms, but its role in alleviating anxiety is limited; video reading (VIR) can partially improve executive function, but its intervention effects on emotional issues are relatively weak. The study results not only validate the effectiveness of multimodal reading interventions but also reveal the specific matching relationship between different media characteristics and cognitive-emotional processing systems, providing theoretical basis and practical guidance for selecting targeted reading intervention methods. Future research could further optimize content design across different reading media and explore the moderating effects of individual differences on intervention outcomes.

## Data Availability

The original contributions presented in the study are included in the article/supplementary material, further inquiries can be directed to the corresponding author/s.
